# A retrospective analysis of the prevalence and impact of associated comorbidities on fibromyalgia outcomes in a tertiary care center

**DOI:** 10.3389/fmed.2023.1301944

**Published:** 2024-01-15

**Authors:** Fernando A. Rivera, Bala Munipalli, Madeleine E. Allman, David O. Hodge, Mikolaj A. Wieczorek, Benjamin Wang, Andy Abril, Adam Perlman, Dacre Knight, Barbara Bruce

**Affiliations:** ^1^Division of General Internal Medicine, Mayo Clinic, Jacksonville, FL, United States; ^2^Department of Psychology, University of Houston, Houston, TX, United States; ^3^Department of Quantitative Health Sciences, Mayo Clinic, Jacksonville, FL, United States; ^4^Division of Rheumatology, Mayo Clinic, Jacksonville, FL, United States; ^5^Department of Psychiatry and Psychology, Mayo Clinic, Jacksonville, FL, United States

**Keywords:** fibromyalgia FM, comorbidities, outcome domains, multimorbidities, treatment

## Abstract

**Background:**

This retrospective study was designed to analyze the prevalence and impact of associated comorbidities on fibromyalgia (FM) outcomes (functionality, pain, depression levels) for patients who participated in an intensive multicomponent clinical program in a tertiary care center.

**Methods:**

Participants included a sample of 411 patients diagnosed with FM at a large tertiary medical center using the 2016 ACR criteria. Patients completed an intensive 2-day cognitive behavioral treatment (CBT) program, filled out the Fibromyalgia Impact Questionnaire Revised (FIQR), the Center for Epidemiologic Studies Depression Scale (CES-D), the Pain Catastrophizing Scale (PCS), and were followed for 6 months after treatment completion. *T*-tests were performed to analyze differences between the presence or absence of select comorbidities for the three outcomes at follow-up. Statistically significant comorbidities (*p* < 0.05) were used as predictors in multivariable logistic regression models.

**Results:**

The FM associated comorbidities in this cohort that had significant impact on the measured outcome domains after treatment program completed were Obesity (FIQR *p* = 0.024), Hypothyroidism (CES-D *p* = 0.023, PCS *p* = 0.035), Gastroesophageal reflux disease GERD (PCS *p* < 0.001), Osteoarthritis (CES-D *p* = 0.047). Interestingly, Headache, the most frequent FM associated comorbidity in this cohort (33.6%), did not have a significant impact on the outcome domains at follow-up. Obesity (18.2%) was the only FM associated comorbidity significantly impacting all three outcome domains at follow-up.

**Conclusion:**

The present study suggests that addressing obesity may significantly impact outcomes in FM patients.

## Introduction

Fibromyalgia (FM) affects approximately 10 million people in the US alone with estimates as high as 8% of the population world-wide (1, 2). The prevalence rate of FM in the general population is estimated to be 2–4% (3). It is a chronic pain syndrome with features that includes widespread musculoskeletal pain, extreme fatigue, sleep disturbance, and cognitive complaints (4–7). FM can result in significant incapacity with 35% of patients with FM in the United States on government disability assistance (8). Patients with FM struggle with depression with numerous studies documenting a high prevalence of depression in this population (9). Further, pain catastrophizing has been linked to important outcomes in chronic pain patients such as higher opioid use, longer hospital stay and increased likelihood of being on disability (10). FM is associated with other medical and/or psychiatry conditions that affect its clinical presentation, and result in complications that require specific treatments, and may potentially impact outcome (11).

FM is considered a complex condition that affects the way that the brain processes pain signal, leading to high sensitivity and discomfort. Along with pain, individual with FM experience sleep disturbances, cognitive difficulties (sometimes refer to as a “fibro fog”), mood disorders. The most widely used criteria for diagnosing FM is the 2016 American College of Rheumatology (ACR) criteria. According to this criteria, widespread pain, tender trigger points,

and the updated comprehensive evaluation symptoms and their impact on a person quality of life are the main elements to make a FM diagnosis currently (3, 5, 6, 12).

Sex and age can play a role in the prevalence, symptoms, and management of FM, though individual experiences can vary. FM is more commonly diagnosed in women than in men. Studies suggest that women have a higher likelihood of developing FM (70–90%). FM can affect individuals of all ages, but it is more commonly diagnosed in middle aged adults (30–50-year-old) (1, 2).

FM patients experience a higher rate of comorbid conditions, specifically rheumatologic and psychiatric conditions as compared with the general population (13–15), that have included coronary artery disease, myocardial infarction (MI), hypertension, stroke (16–18), diabetes mellitus, and irritable bowel syndrome (13, 19). In one study involving a large sample of community-dwelling adults with FM, over 50% were found to have seven or more chronic conditions (20). Most scientific studies that have examined comorbidity in FM has focused on clinical presentation, pain management, and medications (21–24). FM has been associated with various comorbid entities including medical and psychiatric disorders. During the last few decades, the prevalence of chronic illnesses has risen due to multiple factors including but not limited to better understanding of the diseases, increased longevity, improved access to health care, electronic medical record, patient online services, etc. (25, 26). Comorbidities may lead to a delay in the diagnosis of FM, may be mistaken as poor control of the primary disease, may cause leading to incorrect treatment decisions, and may also increase Morbi and mortality in these patients (23, 27).

The aim of this study is to determine the prevalence of comorbidities in a cohort of FM patients (25, 28, 29) who attended a 2-day cognitive behavioral treatment program, and examine the impact of each comorbidity on specific outcomes of level of psychological distress/depression, functional status, and cognitive strategies that specifically impact pain and its management.

## Methods

### Participants and procedure

Participants were recruited from a large tertiary medical center’s Fibromyalgia Treatment Program. Inclusion criteria for the study included a diagnosis with FM using the 2016 ACR criteria (5) and informed consent to participate in the study. No exclusion criteria were used for the study. The Fibromyalgia Treatment Program is an intensive 2-day cognitive behavioral treatment program. Participants completed follow up surveys approximately 6-months following treatment to assess outcomes. The study was approved by the Mayo Clinic Institutional Review Board (Protocol ID: 19–000495). Baseline data including study measures and demographic information were collected before the patients started the 2-day program. Post-intervention data were collected with mailed surveys at post-treatment. If patients did not respond to the mailed survey a reminder letter was sent out.

### Measures

#### Comorbidities

Presence of a comorbidity was defined as any diagnosis from a patient’s medical history. Individual comorbidities were grouped into twelve broader categories with patients belonging to a particular category if they had any diagnosed conditions from medical history or current visit (29, 30). Multi-morbidities were defined as low (2 or less), and high (3 or more) comorbidity-associated medical diagnosis documented at any time (medical history/current visit) (26, 31).

The Fibromyalgia Impact Questionnaire-Revised (FIQR) measures functional status and is the most widely used measure of functional impairment in FM patients. Three domains are evaluated with this measure and include function, overall impact, and symptoms. The scale includes twenty-one items that are scored from 0–10 with higher scores reflecting greater functional impairment. Severe functional impairment is indicated by scores of 60 or above. The psychometric properties of this scale have been researched (12).

The Center for Epidemiologic Studies of depression Scale (CES-D) is a 20-item scale that assesses the presence and severity of depressive symptoms (32). Scores range from 0 to 6-with higher scores reflecting a higher degree of depressive symptomatology. The clinical threshold for depression is a score of 16 on this measure. The psychometric properties of the CES-D are well researched (33).

The Pain Catastrophizing Scale (PCS) is a self-report questionnaire designed to measure the tendency of individuals to catastrophize or magnify the significance of pain they experience. It assesses the degree to which a person experiences some negative thoughts and emotions related to pain, such as rumination, magnification, and helplessness. These 3 subscales help researchers and healthcare professionals better understand and evaluate the psychological impact of pain on individuals (34–36).

### Treatment program

The Fibromyalgia Treatment Program is a 16-h, cognitive behavioral based, group program that addresses education and evidence-based strategies to decrease central sensitization. Strategies addressed include relaxation training, use of moderation, pacing, exercise, cognitive skills, sleep hygiene, stress management, social support, and the use of pharmacological options to improve symptoms and functioning (37). The effectiveness of this intervention has been described elsewhere (38).

### Statistical analysis

Cohort characteristics, comorbidities, and outcomes were summarized with frequency (percentage) for categorical variables and with mean, standard deviation, and range for continuous variables. Twelve individual conditions selected based on prevalence as well as twelve grouped comorbidities were further used in outcome analysis. *T*-tests were performed to analyze differences between presence or absence of individual comorbidities as well as grouped comorbidities for the three outcomes (FIQR, CES-D, PCS at follow-up). Additionally, we compared patients with 2 or less vs. 3 or more comorbidities for each outcome. The comorbidities that were statistically significant comorbidities (*p* < 0.05) in our univariate analyses were selected for inclusion into our multivariable logistic regression models as predictors; all the comorbidities were evaluated under this criterium for each outcome independently. The multivariable logistic regression models were adjusted for patient’s age and sex, where odds ratios (ORs) and 95% confidence intervals (Cis) were estimated; binary outcomes were defined as severe functioning (FIQR >60), severe depression (CES-D ≥ 20), and severe pain catastrophizing (PCS ≥ 30). Analyses were conducted using R Statistical Software (version 4.0.3; R Foundation for Statistical Computing, Vienna, Austria).

The effect of baseline measures of pain, depression, and function was not considered in the regression modeling. The main aim of this study was to look at associations of comorbidities with our outcomes at follow-up. Another study we published using the same cohort discussed the treatment effectiveness between our baseline and follow-up outcome scores (38).

## Results

Participants included 411 patients diagnosed with fibromyalgia at a large tertiary medical center using the 2016 ACR criteria (5). The sample completed an intensive 2-day cognitive behavioral treatment program and were followed after treatment to assess treatment effectiveness. Follow up data was received 4–8 months after treatment with an average for the sample of 6-month follow-up.

**Figure 1:**
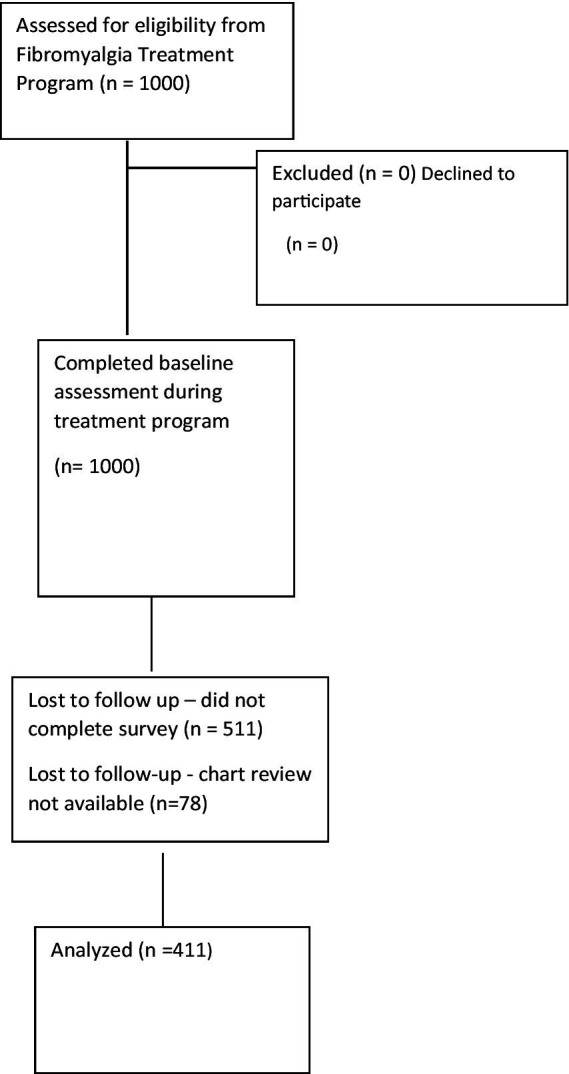
Participant flow chart.

In our cohort, women represent 90.3%, and the age mean was 54.7 years for the overall sample (411) with a standard deviation SD of 13.9 years (Table 1). As seen in Figure 1, 1,000 patients were assessed for eligibility from the Fibromyalgia Treatment Program. From them, zero patients were excluded due to ineligibility and all consented to participate. All 1,000 patients completed baseline data collection. Of them, 511 patients were lost to follow up as they did not complete mailed surveys at 6-months post-intervention. A subset of patients (*n* = 78) were excluded due to inability to locate sufficient medical chart review information. In the present analysis, 411 patients were included.

**Table 1:** Summary of cohort characteristics.

Characteristic	Overall (*N* = 411)
Age at survey (years)
	Mean (SD)	54.7 (13.9)
Range	22.5–85.6
Gender
	Female	371 (90.3%)
Male	40 (9.7%)
Race
	White	372 (90.5%)
Black or African American	16 (3.9%)
Other	23 (5.6%)
Ethnicity
	Hispanic or Latino	34 (8.3%)
Not Hispanic or Latino	368 (89.8%)
Other	8 (2.0%)
Marital Status
	Married/Domestic partnership	300 (73.2%)
Single/Divorced/Widowed	110 (26.8%)
Duration of symptoms
	Less than 1 year	20 (4.9%)
1–2 years	81 (19.7%)
3–5 years	109 (26.5%)
Greater than 5 years	201 (48.9%)
Time since diagnosis (months)
	Mean (SD)	48.1 (79.6)
Range	0.0–480.0
Abdominal pain	41 (10.0%)
Headache	138 (33.6%)
Hypertension	95 (23.1%)
IBS	61 (14.8%)
Insomnia	92 (22.4%)
Low back pain	74 (18.0%)
Osteoarthritis	96 (23.4%)
Obesity	75 (18.2%)
OSA	68 (16.5%)
RA	24 (5.8%)
GERD	17 (4.1%)
Hypothyroidism	29 (7.1%)
Multi-morbidities
	2 or less	276 (67.2%)
3 or more	135 (32.8%)
Admission FIQR
	Mean (SD)	59.1 (18.1)
Range	0.0–94.7
Admission CES-D
	Mean (SD)	25.3 (9.8)
Range	0.0–52.0
Admission PCS
	Mean (SD)	22.8 (12.3)
Range	0.0–52.0
Follow-up FIQR
	Mean (SD)	46.2 (21.3)
Range	0.0–98.0
Follow-up CES-D
	Mean (SD)	21.4 (9.8)
Range	0.0–56.0
Follow-up PCS
	Mean (SD)	15.7 (12.7)
Range	0.0–52.0
Met severe threshold for functional impairment (FIQR >60)	117 (28.5%)
Met clinical threshold for depression (CESD ≥ 20)	235 (57.2%)
Met severe threshold for pain catastrophizing (PCS ≥ 30)	67 (16.3%)

IBS, Irritable Bowel Syndrome; OSA, Obstructive Sleep Apnea; RA, Rheumatoid Arthritis; GERD, Gastroesophageal Reflux Disease; FIQR, Fibromyalgia Impact Questionnaire Revised; CES-D, Center for Epidemiologic Studies Depression Scale; PCS, Pain Catastrophizing Scale.

The results, as contained in Table 1, revealed the following comorbidities: headaches (33.6%), osteoarthritis (23.4%), hypertension (23.1%), insomnia (22.4%), obesity (18.2%), low back pain (18%), obstructive sleep apnea (OSA) (16.5%), irritable bowel syndrome (IBS) (14.8%), abdominal pain (10%), hypothyroidism (7.1%), rheumatoid arthritis (RA) (5.8%), and gastroesophageal reflux (GERD) (4.1%).

Comparisons between patients with vs. patients without a certain comorbidity for the three outcomes are summarized in Table 2. Obesity negatively impacted functioning at follow-up with obese patients having, on average, a poorer functioning score than non-obese patients (mean: 51.0 vs. 45.1, *p* = 0.024). Patients with hypothyroidism, however, were more likely to have lower depression (mean: 18.2 vs. 21.6, *p* = 0.023) and pain catastrophizing (mean: 12.1 vs. 16.0, *p* = 0.035) scores at follow-up than patients without hypothyroidism. Similarity, patients with osteoarthritis had a lower depression score (mean: 19.6 vs. 21.9, *p* = 0.047) and patients with gastroesophageal reflux (GERD) had a lower pain catastrophizing score (mean: 12.1 vs. 16.0, *p* = 0.023) six months after the treatment program. Although not statistically significant, abdominal pain, headache, irritable bowel syndrome, and rheumatoid arthritis negatively impacted all three outcomes at follow-up (Table 2).

**Table 2:** Impact of select comorbidities on outcomes at follow-up.

Comorbidity type/Outcome	No comorbidity	Comorbidity	*p*-value
Abdominal pain	N = 370	N = 41	
FIQR			0.148
Mean (SD)	45.6 (21.2)	51.0 (22.3)	
Range	0.0–98.0	18.0–96.8	
CES-D			0.169
Mean (SD)	21.1 (9.5)	23.8 (12.0)	
Range	0.0–47.0	4.0–56.0	
PCS			0.496
Mean (SD)	15.6 (12.6)	17.0 (12.9)	
Range	0.0–52.0	0.0–50.0	
Headache	N = 273	N = 138	
FIQR			0.116
Mean (SD)	45.0 (21.4)	48.5 (21.2)	
Range	4.0–98.0	0.0–96.8	
CES-D			0.072
Mean (SD)	20.8 (9.6)	22.6 (10.1)	
Range	0.0–47.0	0.0–56.0	
PCS			0.054
Mean (SD)	14.9 (12.7)	17.4 (12.4)	
Range	0.0–52.0	0.0–50.0	
Hypertension	N = 316	N = 95	
FIQR			0.211
Mean (SD)	45.4 (21.3)	48.6 (21.3)	
Range	0.0–98.0	4.3–92.0	
CES-D			0.989
Mean (SD)	21.4 (9.9)	21.4 (9.5)	
Range	0.0–56.0	2.0–44.0	
PCS			0.477
Mean (SD)	15.5 (12.7)	16.5 (12.5)	
Range	0.0–52.0	0.0–46.0	
IBS	N = 350	N = 61	
FIQR			0.604
Mean (SD)	45.9 (21.2)	47.5 (22.2)	
Range	0.0–96.8	10.7–98.0	
CES-D			0.180
Mean (SD)	21.1 (9.5)	23.1 (11.2)	
Range	0.0–56.0	0.0–45.0	
PCS			0.182
Mean (SD)	15.3 (12.4)	17.9 (14.1)	
Range	0.0–52.0	0.0–50.0	
Insomnia	N = 319	N = 92	
FIQR			0.236
Mean (SD)	46.8 (21.0)	43.7 (22.4)	
Range	4.0–98.0	0.0–88.3	
CES-D			0.855
Mean (SD)	21.3 (9.5)	21.6 (10.8)	
Range	0.0–56.0	0.0–45.0	
PCS			0.990
Mean (SD)	15.7 (12.8)	15.7 (12.3)	
Range	0.0–52.0	0.0–43.0	
Low back pain	N = 337	N = 74	
FIQR			0.494
Mean (SD)	45.8 (21.2)	47.7 (21.9)	
Range	2.0–98.0	0.0–88.7	
CES-D			0.102
Mean (SD)	21.8 (9.8)	19.7 (9.8)	
Range	0.0–56.0	0.0–42.0	
PCS			0.132
Mean (SD)	16.1 (12.9)	13.8 (11.7)	
Range	0.0–52.0	0.0–46.0	
Osteoarthritis	N = 315	N = 96	
Follow-up FIQR			0.528
Mean (SD)	46.5 (21.5)	45.0 (20.8)	
Range	0.0–96.8	4.0–98.0	
Follow-up CES-D			0.047**
Mean (SD)	21.9 (9.6)	19.6 (10.2)	
Range	0.0–56.0	0.0–45.0	
Follow-up PCS			0.424
Mean (SD)	16.0 (12.7)	14.8 (12.7)	
Range	0.0–52.0	0.0–50.0	
Obesity	N = 336	N = 75	
FIQR			0.024**
Mean (SD)	45.1 (21.4)	51.0 (20.3)	
Range	0.0–98.0	8.5–81.8	
CES-D			0.092
Mean (SD)	21.0 (9.9)	23.0 (9.1)	
Range	0.0–56.0	2.0–47.0	
PCS			0.371
Mean (SD)	15.5 (12.8)	16.9 (12.2)	
Range	0.0–52.0	0.0–46.0	
OSA	N = 343	N = 68	
FIQR			0.913
Mean (SD)	46.1 (21.2)	46.4 (22.1)	
Range	0.0–98.0	5.0–88.8	
CES-D			0.611
Mean (SD)	21.5 (9.4)	20.8 (11.5)	
Range	0.0–56.0	0.0–43.0	
PCS			0.921
Mean (SD)	15.7 (12.3)	15.9 (14.6)	
Range	0.0–50.0	0.0–52.0	
RA	N = 387	N = 24	
FIQR			0.188
Mean (SD)	45.8 (21.4)	51.7 (20.6)	
Range	0.0–98.0	7.7–84.8	
CES-D			0.218
Mean (SD)	21.3 (9.8)	23.7 (9.0)	
Range	0.0–56.0	8.0–40.0	
PCS			0.101
Mean (SD)	15.4 (12.5)	20.6 (14.7)	
Range	0.0–52.0	0.0–50.0	
GERD	N = 394	N = 17	
FIQR			0.050**
Mean (SD)	46.6 (21.2)	35.1 (22.1)	
Range	2.0–98.0	0.0–70.2	
CES-D			0.286
Mean (SD)	21.5 (9.7)	18.3 (11.9)	
Range	0.0–56.0	0.0–44.0	
PCS			< 0.001
Mean (SD)	16.0 (12.8)	8.6 (7.0)	
Range	0.0–52.0	0.0–22.0	
Hypothyroidism	N = 382	N = 29	
FIQR			0.527
Mean (SD)	46.3 (21.4)	43.8 (20.2)	
Range	0.0–98.0	4.3–81.3	
CES-D			0.023*
Mean (SD)	21.6 (9.9)	18.2 (7.4)	
Range	0.0–56.0	5.0–33.0	
PCS			0.035*
Mean (SD)	16.0 (12.9)	12.1 (9.0)	
Range	0.0–52.0	0.0–40.0	

*p*-values result from two-sided *t*-test. ** *p* < 0.05; * *p* < 0.01. FIQR, Fibromyalgia Impact Questionnaire Revised; CES-D, Center for Epidemiologic Studies Depression Scale; PCS, Pain Catastrophizing Scale.

In Table 3, we examined logistic regressions for significant comorbidities and binary treatment outcomes. In our multivariate analysis, obesity was the only statistically significant predictor of FIQR score greater than 60 after adjusting for patient’s age and sex, showing that obese patients were twice more likely to have poorer functional status after completing the treatment program (OR = 1.77, 95% CI: 1.04–2.98, *p* = 0.033).

**Table 3:** Comparison between patients with vs. without select comorbidities by outcomes at follow-up.

	OR (95% CI)	*p*-value
FIQR (>60)
Obesity	1.77 (1.04, 2.98)	0.033*
CES-D (≥20)
Osteoarthritis	0.79 (0.49, 1.28)	0.33
Hypothyroidism	0.48 (0.21, 1.04)	0.067
PCS (≥30)
Hypothyroidism	0.36 (0.06, 1.27)	0.18

*p*-values result from multivariate logistic regression models. All the models were adjusted for age and sex. ** *p* < 0.05; *p < 0.01. OR, odds ratio; CI, confidence interval; FIQR, Fibromyalgia Impact Questionnaire Revised; CES-D, Center for Epidemiologic Studies Depression Scale; PCS, Pain Catastrophizing Scale.

Adjusting logistic regression models for age and sex is important for several reasons: confounding variables can distort the observed associations, population characteristics are fundamental demographic variables that reflect important differences in the population, and standardization allows for more meaningful comparisons and reduces the potential impact of demographic differences on the observed associations (3, 6, 8, 39).

Table 4 compares differences between grouped comorbidities for these three outcomes at follow-up. Patients in the psychiatry and gynecology groups tended to have higher depression scores (mean: 22.6 vs. 20.1, *p* = 0.008; 25.2 vs. 21.1, *p* = 0.022, respectively). Also, patients in the psychiatry group experienced higher pain catastrophizing score (mean: 17.1 vs. 14.4, *p* = 0.035).

**Table 4:** Impact of select grouped comorbidities on outcomes at follow-up.

Comorbidity type/Outcome	No comorbidity	Comorbidity	*P*-value
Rheumatological	N = 14	N = 395	
FIQR			0.112
Mean (SD)	36.3 (22.2)	46.5 (21.3)	
Range	10.3–84.8	0.0–98.0	
CES-D			0.078
Mean (SD)	16.7 (9.2)	21.5 (9.8)	
Range	0.0–31.0	0.0–56.0	
PCS			0.077
Mean (SD)	11.1 (8.9)	15.8 (12.8)	
Range	0.0–33.0	0.0–52.0	
Psychiatry	N = 212	N = 197	
FIQR			0.600
Mean (SD)	45.6 (21.1)	46.7 (21.7)	
Range	4.0–98.0	0.0–96.8	
CES-D			0.008*
Mean (SD)	20.1 (9.1)	22.6 (10.3)	
Range	0.0–44.0	0.0–56.0	
PCS			0.035**
Mean (SD)	14.4 (12.0)	17.1 (13.3)	
Range	0.0–50.0	0.0–52.0	
Pain	N = 232	N = 177	
FIQR			0.702
Mean (SD)	45.8 (22.2)	46.6 (20.4)	
Range	2.0–98.0	0.0–92.0	
CES-D			0.737
Mean (SD)	21.2 (9.8)	21.5 (9.7)	
Range	0.0–56.0	0.0–44.0	
PCS			0.602
Mean (SD)	15.4 (12.8)	16.1 (12.6)	
Range	0.0–52.0	0.0–50.0	
Neurological	N = 249	N = 160	
FIQR			0.478
Mean (SD)	45.6 (21.5)	47.1 (21.3)	
Range	4.0–98.0	0.0–96.8	
CES-D			0.405
Mean (SD)	21.0 (9.6)	21.8 (10.1)	
Range	0.0–47.0	0.0–56.0	
PCS			0.551
Mean (SD)	15.4 (13.0)	16.1 (12.3)	
Range	0.0–52.0	0.0–50.0	
Endocrinological	N = 281	N = 128	
FIQR			0.662
Mean (SD)	45.9 (21.8)	46.8 (20.6)	
Range	0.0–98.0	4.3–87.0	
CES-D			0.518
Mean (SD)	21.1 (10.0)	21.8 (9.3)	
Range	0.0–56.0	0.0–47.0	
PCS			0.413
Mean (SD)	16.0 (13.1)	15.0 (11.7)	
Range	0.0–52.0	0.0–46.0	
Cardiovascular	N = 292	N = 117	
FIQR			0.320
Mean (SD)	45.5 (21.3)	47.8 (21.6)	
Range	0.0–98.0	4.3–92.0	
CES-D			0.715
Mean (SD)	21.2 (9.9)	21.6 (9.6)	
Range	0.0–56.0	2.0–44.0	
PCS			0.455
Mean (SD)	15.4 (12.7)	16.4 (12.8)	
Range	0.0–52.0	0.0–46.0	
Pulmonary	N = 308	N = 101	
FIQR score			0.485
Mean (SD)	45.7 (21.5)	47.4 (21.0)	
Range	0.0–98.0	5.0–88.8	
CES-D			0.597
Mean (SD)	21.5 (9.5)	20.9 (10.5)	
Range	0.0–56.0	0.0–43.0	
PCS			0.718
Mean (SD)	15.5 (12.4)	16.1 (13.7)	
Range	0.0–50.0	0.0–52.0	
Sleep	N = 309	N = 100	
FIQR			0.108
Mean (SD)	47.2 (21.0)	43.1 (22.3)	
Range	4.0–98.0	0.0–88.3	
CES-D			0.954
Mean (SD)	21.3 (9.5)	21.3 (10.7)	
Range	0.0–56.0	0.0–45.0	
PCS			0.646
Mean (SD)	15.8 (12.9)	15.2 (12.1)	
Range	0.0–52.0	0.0–43.0	
Gastrointestinal	N = 310	N = 99	
FIQR			0.564
Mean (SD)	45.8 (20.9)	47.3 (22.8)	
Range	2.0–88.8	0.0–98.0	
CES-D			0.438
Mean (SD)	21.1 (9.1)	22.1 (11.7)	
Range	0.0–47.0	0.0–56.0	
PCS			0.262
Mean (SD)	15.3 (12.4)	17.0 (13.6)	
Range	0.0–52.0	0.0–50.0	
Muscle-skeletal	N = 313	N = 96	
FIQR			0.810
Mean (SD)	46.0 (21.3)	46.6 (21.9)	
Range	2.0–96.8	0.0–98.0	
CES-D			0.064
Mean (SD)	21.8 (9.7)	19.7 (9.9)	
Range	0.0–56.0	0.0–44.0	
PCS			0.116
Mean (SD)	16.2 (13.0)	14.0 (11.6)	
Range	0.0–52.0	0.0–50.0	
Gynecology	N = 388	N = 21	
FIQR			0.290
Mean (SD)	45.9 (21.2)	51.7 (24.0)	
Range	0.0–98.0	4.3–93.5	
CES-D			0.022*
Mean (SD)	21.1 (9.9)	25.2 (7.3)	
Range	0.0–56.0	10.0–37.0	
PCS			0.087
Mean (SD)	15.5 (12.7)	19.9 (11.0)	
Range	0.0–52.0	0.0–39.0	
Nephrology	N = 394	N = 15	
FIQR			0.058
Mean (SD)	45.8 (21.3)	56.8 (20.4)	
Range	0.0–98.0	18.0–86.3	
CES-D			0.294
Mean (SD)	21.2 (9.8)	23.6 (8.2)	
Range	0.0–56.0	10.0–39.0	
PCS			0.541
Mean (SD)	15.6 (12.6)	18.1 (15.1)	
Range	0.0–52.0	4.0–48.0	

*p*-values result from two-sided *t*-test. ** *p* < 0.01; * *p* < 0.05. FIQR, Fibromyalgia Impact Questionnaire Revised; CES-D, Center for Epidemiologic Studies Depression Scale; PCS, Pain Catastrophizing Scale.

Comparison was done with those having 2 or fewer comorbidities vs. 3 or more comorbidities to see if the outcome measures were negatively impacted by the number of comorbidities (possibly indicative of worse health which could be confounding or contributing factors to dysfunction, depression, and pain catastrophizing) in addition to the condition of FM. In our cohort, 32.8% of patients had 3 or more comorbidities while 67.2% had 2 or less (Table 1). When we analyzed impact of multi-morbidities on outcomes, we found no statistically significant differences between the two groups in functioning, depression or pain catastrophizing scores (Table 5).

**Table 5:** Impact of multi-morbidities on outcomes at follow-up.

	2 or less (*N* = 276)	3 or more (*N* = 135)	*P*-value
FIQR			0.486
Mean (SD)	45.6 (21.1)	47.2 (21.9)	
Range	2.0–98.0	0.0–92.0	
CES-D			0.993
Mean (SD)	21.4 (9.4)	21.4 (10.6)	
Range	0.0–56.0	0.0–45.0	
PCS			0.669
Mean (SD)	15.5 (12.6)	16.1 (12.9)	
Range	0.0–52.0	0.0–48.0	

*p*-values result from two-sided *t*-test. **p < 0.01;* *p* < 0.05. FIQR, Fibromyalgia Impact Questionnaire Revised; CES-D, Center for Epidemiologic Studies Depression Scale; PCS, Pain Catastrophizing Scale.

## Discussion

The present study assessed the prevalence and impact of comorbidities on treatment effectiveness in FM patients. We examined comorbidities and treatment outcomes (functional impairment, pain catastrophizing, and depression) using several statistical techniques. The data revealed that in our sample, similar to other published studies with FM populations, the most common comorbid conditions were identified: headaches, hypertension, osteoarthritis, and insomnia. Unlike other published studies with higher rates, Rheumatoid arthritis was only identified in 5.8% of our sample (12, 21–37).

Treatment outcomes were then compared for FM patients with and without each comorbidity. This analysis indicated that FM patients with obesity had significantly poorer treatment outcomes in functional impairment. Other comorbidities, including Osteoarthritis, GERD, and Hypothyroidism appeared to impact treatment outcomes in a positive direction. These positive findings may be artificial, as the comorbidity occurred in a very small subset of patients (e.g., Osteoarthritis *n* = 96; GERD *n* = 17; Hypothyroidism *n* = 29).

Taking into account age and sex, Odds Ratios (ORs) were analyzed for Obesity, Osteoarthritis, and Hypothyroidism. These comorbidities were selected based on their significance in the previous analysis (40, 41). ORs were calculated to predict dichotomous treatment outcomes (FIQR >60 = significantly impaired; CES-D > 20 = clinically significant depression; PCS > 30 = clinically significant pain catastrophizing). Of these OR’s, only Obesity indicated a significantly increased risk of functional impairment (OR = 1.77). Put differently, FM patients with Obesity had a 77% higher risk of being significantly impaired at 6-months follow-up, compared to FM patients without Obesity. Obesity adversely affected functioning in our sample at follow-up and needs further investigation. Obesity may impact ability to exercise leading to a deconditioned state that further intensifies FM symptoms (Table 3). A previous systematic review and meta-analysis showed that obesity may impact FM in several ways in addition to potentially limiting exercise or activity level which included level of pain, number of tender trigger points used in previous diagnostic criteria, level of disability, degree of fatigue, and sleep disturbance as well as quality of life (42).

We then grouped comorbidities into different specialty domains (e.g., Rheumatological, Psychiatry, Pain). These analyses revealed Psychiatric comorbidities yielded associations with increased depression and pain catastrophizing scores at follow-up. As these outcomes are expected to correlate with psychological functioning, this finding is expected. The prevalence of psychiatric disorders found in our study was 48.2% which is significantly higher than a previous report where only 25.3% were found to have a psychiatric disorder (27). However, Kleycamp et al. (43) completed a systematic overview of psychiatric and chronic pain comorbidities among patients diagnosed with FM and noted the most prevalent comorbidity was depression that was found in over 50% of patients similar to our findings. Depression (38) is a significant comorbidity associated with FM and prevalence was consistent with results of another cross-sectional studies (44) In addition, Gynecological comorbidities were associated with worse depressive symptoms at follow-up. Gynecological comorbidities were less frequently observed in this study sample (*n* = 21). This finding is consistent with other studies with depression and Gynecological conditions.

Finally, we examined the impact of multimorbidities on treatment outcomes. Data revealed that the Fibromyalgia Treatment Program was equally efficacious for patients with 2 or less comorbidities as 3 or more comorbidities. To clarify the possible confusion between what constitutes a FM symptom versus an associated comorbidity, we based this not only in terms of the scientific society criteria, but also in the consideration that pain syndrome is not exclusive of FM, it is one of its main characteristics. Abdominal pain, headache could be part of the FM diagnosis, however there are other areas to be considered such as overlapping chronic pain syndromes that make them sometimes independent conditions (19–23). We included abdominal pain, headache since they were documented in the electronic medical record prior to the diagnosis of FM, and specific treatments and follow ups were given previously.

Insomnia and sleep disorder are related but have distinct differences. FM symptoms could include sleep disturbance; however, this is a broader term that encompasses various conditions that disrupt normal sleep pattern. Sleep disorders can include insomnia, as well as other conditions like obstructive sleep apnea (OSA), narcolepsy, restless leg syndrome, and parasomnias. Each sleep disorder has his own specific characteristics diagnostic criteria (42,44,45). We have included insomnia as an associated comorbidity in this study since prior to the diagnosis of FM, and documented in medical history, it was a diagnosis (22.38%), not a FM symptom and a specific treatment and follow ups were offered in some of the cohort patients.

Limitations of this study include the setting of a tertiary medical care clinic that results in patients with a higher degree of symptom severity and potentially comorbidity. Additionally, conducting multiple comparisons for hypothesis-generation purposes increases the risk of false positive results. This possibility should be considered when interpreting the findings. Moreover, correction procedures for multiple comparisons were not applied in our study, potentially influencing the significance of certain findings. Future research should address this limitation by employing appropriate correction methods. Further replication studies are needed to validate the significant finding related to obesity and establish the reliability and generalizability of our results.

One of the major limitations of this study is that the comorbidities were collected from medical records and relied on a condition having been diagnosed/documented in the patient notes to be considered a comorbidity The process of collecting data from the electronic medical record was done through an electronic search and by hand in order to ensure accuracy of the data. However, these methods used retrospectively may have underestimated the occurrence of comorbid health conditions. Future studies may be able to collect this information from health care providers prospectively to improve this aspect of the data. Given the limited sample size specific to our tertiary care setting, it is unclear whether it is the obesity or a combination of other less statistically significant comorbidities in association with the obesity that negatively impacted these outcome measures along with the FM. The study assessed the prevalence of comorbidities in our FM patients, but further evaluation needs to be done regarding the generalizability of our results. Further, in our population of FM patients, headaches, osteoarthritis, and hypertension were the most common comorbidities and although cannot be directly linked to gender or age, they are common concerns in middle-aged women (90% of our FM population). Lastly, the limited sample size may impact the percentage of FM patients with concomitant rheumatoid arthritis compared to other studies.

The most important clinical implication of this study is that obesity, which is a modifiable comorbidity, had a significant influence on treatment outcomes. In other words, treatment of obesity in tandem with treatment of FM may yield improved patient outcomes compared to treatment of FM alone for patients with comorbid obesity.

## Conclusion

.Our data suggests that specific comorbidities may need to be addressed more aggressively by primary and specialty physicians to improve outcomes in FM. In particular, obesity was found to negatively impact the success of an intense cognitive behavioral treatment program and needs further investigation. This finding has important implications in the treatment of FM as obesity has been linked to many other modifiable comorbid conditions. Healthcare providers who take care of these patients should keep in mind the potential impact of associated comorbidities on outcomes and address and modify them if possible.

## Data availability statement

The original contributions presented in the study are included in the article/supplementary material, further inquiries can be directed to the corresponding author.

## Ethics statement

The studies involving humans were approved by Mayo Clinic Institutional Review Board. The studies were conducted in accordance with the local legislation and institutional requirements. The participants provided their written informed consent to participate in this study.

## Author contributions

FR: Conceptualization, Writing – original draft, Writing – review & editing, Methodology, Project administration. BM: Conceptualization, Writing – original draft, Writing – review & editing. MA: Conceptualization, Methodology, Writing – review & editing. DH: Conceptualization, Data curation, Formal analysis, Investigation, Methodology, Writing – review & editing. MW: Conceptualization, Data curation, Formal analysis, Investigation, Writing – review & editing. BW: Conceptualization, Methodology, Writing – review & editing. AA: Conceptualization, Methodology, Writing – review & editing. AP: Conceptualization, Methodology, Writing – review & editing. DK: Conceptualization, Methodology, Writing – review & editing. BB: Conceptualization, Investigation, Methodology, Writing – original draft, Writing – review & editing.
